# Factors associated with grade 1 hypertension: implications for hypertension care based on the Dietary Approaches to Stop Hypertension (DASH) in primary care settings

**DOI:** 10.1186/s12875-015-0239-4

**Published:** 2015-02-27

**Authors:** Harry HX Wang, Martin CS Wong, Rosina Y Mok, Mandy WM Kwan, Wai Man Chan, Carmen KM Fan, Catherine LS Lee, Sian M Griffiths

**Affiliations:** JC School of Public Health and Primary Care, Faculty of Medicine, The Chinese University of Hong Kong, Shatin, New Territories Hong Kong; School of Public Health, Sun Yat-Sen University, Guangzhou, 510080 People’s Republic of China; General Practice and Primary Care, Institute of Health and Wellbeing, University of Glasgow, Glasgow, G12 9LX UK; Family Medicine and Primary Health Care, Hospital Authority, Hong Kong, Hong Kong; Food and Environmental Hygiene Department, Hong Kong, Hong Kong

**Keywords:** Grade 1 hypertension, Risk factors, Dietary approaches to stop hypertension, DASH, Reference framework, Community medicine, Primary care

## Abstract

**Background:**

A Reference Framework for Hypertension Care was recently developed by Hong Kong government to emphasise the importance of primary care for subjects with high blood pressure (BP). The Dietary Approaches to Stop Hypertension (DASH) interventional regime was recommended for patients aged 40–70 years with grade 1 hypertension (having systolic BP of 140-159 mmHg and/or diastolic BP of 90-99 mmHg). This study explored factors associated with grade 1 hypertension among subjects screened in primary care settings.

**Methods:**

The study sample consisted of community dwellers (N = 10,693) enrolled in a primary care programme in which participants overall had similar characteristics when compared to the Hong Kong population census. Invitation phone calls were given by trained researchers to a randomly selected subjects (N = 2,673, [50% of total subjects aged 40–70 years]) between January and June 2013. BP and body mass index (BMI) were measured by trained clinical professionals according to a standard protocol. Interviewer-administered survey questionnaires were used to collect self-report information on socio-demographics, family history, and lifestyle characteristics. Multiple logistic regression analysis was performed to explore factors associated with grade 1 hypertension. Adjusted odds ratios (aORs) were estimated with 95% confidence intervals (CI).

**Results:**

A total of 679 out of 2,673 subjects agreed to participate in the screening and completed the baseline assessment (100% completion rate), among which, 320 subjects (47.1%, [320/679]) were grade 1 hypertensive. Unhealthy diet (aOR = 2.19, 95%CI 1.04-4.62), irregular meals (aOR = 1.47, 95%CI 1.11-1.95), BMI >27.5 kg/m^2^ (aOR = 1.87, 95%CI 1.53-2.27), duration of cigarette smoking (aOR = 1.83 per year), increased daily cigarette consumption (aOR = 1.59 per pack [20 cigarettes per pack]), duration of alcohol drinking (aOR = 1.65 per year), and higher frequency of weekly binge drinking (aOR = 1.87 per occasion) were independently associated with grade 1 hypertension. The increase in the number of risk factors combined significantly correlated with higher predicted probability of grade 1 hypertension.

**Conclusions:**

Dietary-intake factors were significantly associated with grade 1 hypertension, echoing the recommendation in the Reference Framework on incorporating dietary-related intervention based on the DASH approach for hypertension care in primary care settings. The association between aggregate risk factors and grade 1 hypertension should also be taken into consideration in long-term preventive strategy.

## Background

High blood pressure (BP) is one of the most known independent risk factors for cardiovascular and cerebrovascular diseases. More than one-fourth of the adult population worldwide have systolic BP (SBP)/diastolic BP (DBP) persistently over 140/90 mmHg [1], and the prevalence of hypertension is predicted to increase by 42% in 2025 [2]. In Hong Kong where the Chinese population are predominant, a recent territory-wide population-based health survey suggested that hypertension was the second most prevalent chronic condition [3]. However, it is believed that hypertension rarely presented clear symptoms until BP goes extremely high with concomitant complications. Many patients might not be fully aware of the presence of hypertension until being screened with on-site physical measurement, as shown in a previous study that 15.1% of diagnosed hypertensive patients in Hong Kong were newly identified by field measurement [4]. Hypertension increases health care utilisation particularly in primary care where hypertension serves as a major reason for doctor consultation [5].

International guidelines [1,6-8] has classified hypertension into three different classes, i.e., grade 1 hypertension (SBP of 140-159 mmHg and/or DBP of 90-99 mmHg); or grade 2 hypertension (SBP of 160-179 mmHg and/or DBP of 100-109 mmHg); or grade 3 hypertension (SBP ≥180 mmHg and/or DBP ≥110 mmHg). Many clinical trials in primary care settings focused on the effectiveness of drug therapies in grade 2/3 hypertensive patients. However, pharmacological treatments could lead to escalating cost of drugs, complex multi-drug interactions, and thus suboptimal adherence with drug prescriptions and uncontrolled hypertension. It has been recommended that adults aged 40–70 years should be screened for the risk of cardiovascular disease and received regular follow up visit when necessary [9]. A Reference Framework for Hypertension Care for Adults in Primary Care Settings [10] was recently developed by the Hong Kong government in 2010, which advocated the adoption of the Dietary Approaches to Stop Hypertension (DASH) eating plan particularly among subjects with grade 1 hypertension aged between 40–70 years old who were not known to have cardiovascular disease or to be at high risk of cardiovascular disease [11-14]. However, to our knowledge, no study has been devoted to translating the Reference Framework into real primary care settings [4]. Some earlier literature in the Western population has pointed out that several factors such as ageing, higher body mass index (BMI), tobacco smoking, family history, and physical inactivity could largely contribute to the risk of hypertension overall [15]. Nevertheless, few studies have focused on the progression from normotensive status to grade 1 hypertension in the East particularly in the Chinese population. Knowledge on the effects of dietary factors and quantified lifestyle behaviours such as the amount of smoking and drinking on the progression to grade 1 hypertension are largely inadequate. It is possible that proactive screening of grade 1 hypertension in primary care followed by less expensive methods such as lifestyle modifications to reduce BP in this early phase of disease progression may convey greater long-term benefits [16,17]. Therefore, more information is needed on plausible determinants and dietary factors particularly associated with grade 1 hypertension to inform front-line primary care physicians in the community for effective hypertension prevention in real practice.

This study followed the Reference Framework to screen subjects with grade 1 hypertension in primary care settings, and explored factors associated with grade 1 hypertension.

## Methods

### Study design

**Figure 1:**
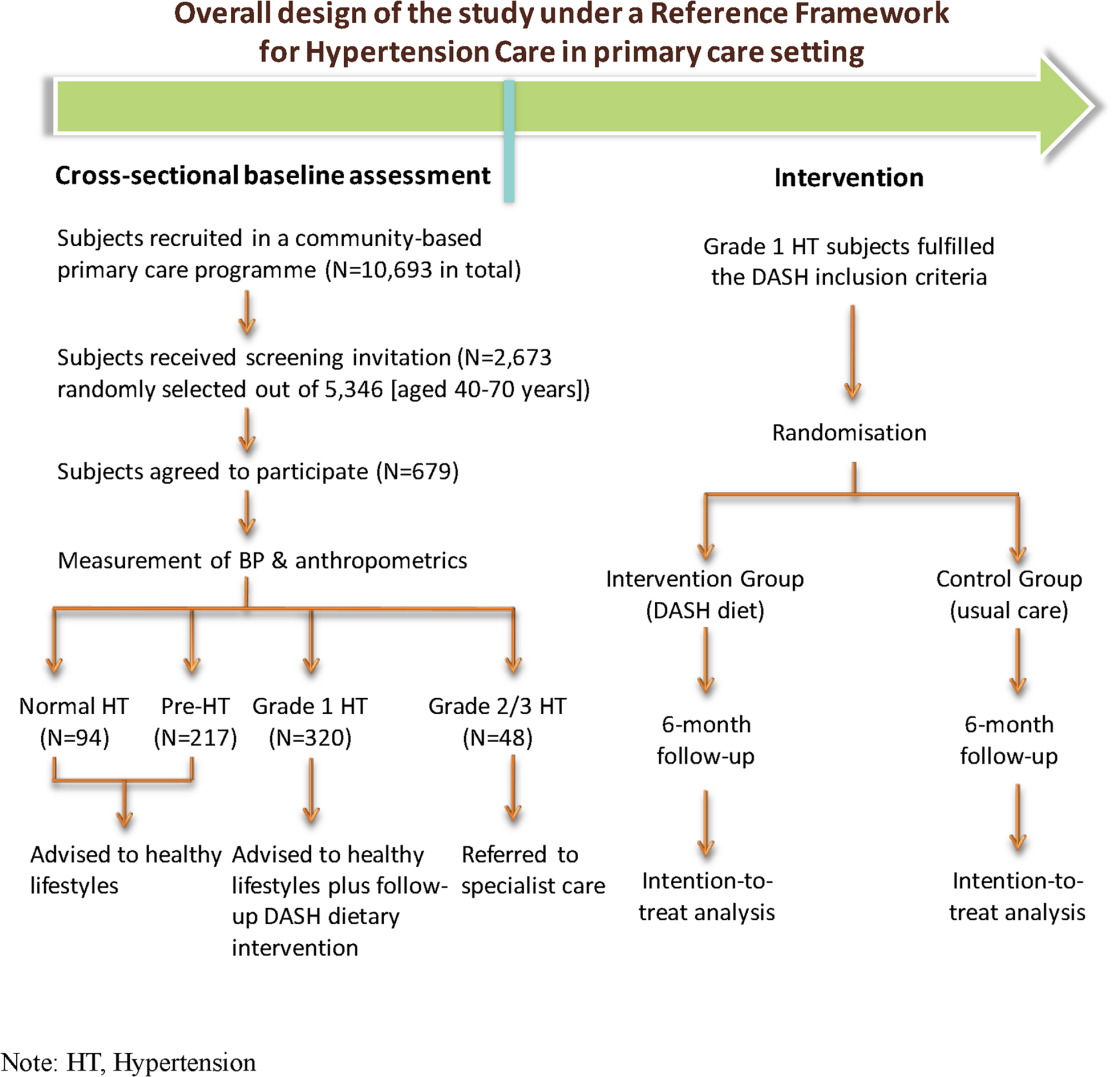
**Study flowchart.**

This study was a baseline assessment of a larger prospective intervention cohort study which evaluated the effectiveness of DASH module in the Reference Framework among grade 1 hypertensive subjects in primary care settings. The study was conducted between January and June 2013 in one geographic region in Hong Kong where the predominant population are Chinese. All subjects were community dwellers previously enrolled in a primary care programme (N = 10,693 in total) launched by the Family Medicine Clinic, Lek Yuen Health Centre in New Territories East Cluster, Hong Kong. The programme participants overall had similar characteristics when compared to the Hong Kong population census [18], in terms of age (40.6 versus 41.7 years on average), female gender (51.5% versus 53.3%), education level (83.4% versus 77.1% for those completed lower secondary or above education), and employment status (83.2% versus 88.8% for having employment). Invitation phone calls by trained researchers were given to 50% of all subjects aged 40–70 years [N = 2,673 out of 5,346] selected by simple randomisation. Onsite physical examinations by trained clinical professional were conducted among those who agreed to participate in the screening (N = 679, participation rate of 25.4% [679/2,673]). All participants were interviewed with questionnaires by trained interviewers for data collection. The overall study flow chart was shown in Figure 1.

### Measurement of BP and anthropometric parameters

BP values were measured using an automated sphygmomanometer in patient’s right arm with an appropriately sized cuff by trained clinical staffs according to a standard protocol [1]. Patients were required to rest in a sitting position for at least five minutes before BP measurements. All patients had BP measured at least one hour after their last meal, and at least 30 minutes after tobacco smoking or consumption of alcohol or caffeinated beverages. The first and fifth Korotkoff sounds were recorded as Systolic BP (SBP) and Diastolic BP (DBP), respectively. BP was measured three times, and the mean of BP readings was used. Normotensive BP was defined as having SBP <120 mmHg and DBP <80 mmHg with no antihypertensive medication taking. Pre-hypertension was defined as having SBP of 120–139 mmHg and/or DBP of 80–89 mmHg, and grade 1 hypertension was defined as having SBP of 140–159 mmHg and/or DBP 90–99 mmHg. Grade 2 or grade 3 hypertension was defined as SBP ≥160 mmHg and/or DBP ≥100 mmHg and/or use of antihypertensive medication in the past two weeks. Weight was measured with light clothing and without shoes by a calibrated scale and height was measured using a wall-mounted stadiometer. The body mass index (BMI) was calculated as weight (in kilograms) divided by the square of the height (in metres). The recommendation of BMI cut-off points for overweight and obesity in Asian populations was used to categorise BMI as underweight (<18.5 kg/m^2^), increasing but acceptable risk (18.5-22.9 kg/m^2^), increased risk (23–27.5 kg/m^2^), or high risk (>27.5 kg/m^2^) [19].

### Patients

The inclusion criteria of the DASH intervention in the Reference Framework was followed to screen eligible study participants who were Chinese adults aged between 40 to 70 years. All participants provided written informed consent for the participation in the screening and interviewer-administered survey. Following the Reference Framework, advices on adopting healthier lifestyles were given to patients with no elevated BP or with pre-hypertension. Patients with grade 1 hypertension received advices on healthy lifestyles plus follow-up DASH dietary intervention, and those with grade 2 or grade 3 hypertension were referred to specialist care (Figure 1).

### Data collection

Data collection was conducted by one physician (RYM) and one clinical researcher (CKFM) trained by the Principal Investigator (MCSW). This resembled the real practice of home-visit health care usually delivered by one physician plus one nurse. Onsite physical examination anthropometric measurements were conducted (by RYM) to collect baseline data on BP, weight, and height. Interviewer-administered questionnaires were used (by CKFM) to obtain information on socio-demographic characteristics, lifestyle factors (physical exercises, drinking, smoking, and diet pattern), and parental history of hypertension (whether father or mother was diagnosed with hypertension). Physical exercises referred to vigorous physical exercises in which the heart beats faster and the breathing is heavier for at least 20 minutes in leisure time every week regularly. Subjects were also asked about the duration of cigarette smoking and the average amount of cigarette consumption per day, according to the number of packs smoked per day (20 cigarettes per pack). Subjects who had consumed alcohol in the previous 12 months (excluding unusual or accidental consumption of alcohol) were considered as current drinkers, and were asked about the duration of alcohol drinking. We followed the methods used in our previous study in Hong Kong to quantify one serving of alcohol as “a can of beer or a 5-ounce glass of wine, equivalent to approximately half a can of soda, or a shot of hard liquor/spirits, equivalent to one-eighth of a can of soda [20]”. We also asked the frequency of weekly binge drinking, i.e., having five servings of alcohol (for males) or four servings (for females) on one occasion [20]. The assessment on dietary pattern was based on the servings sizes recommended for 8 food groups (grains; vegetables; fruits; dairy; meat, poultry, fish, and eggs; nuts, seeds, and legumes; fats and oils; sweets) in the DASH eating plan [21]. For each group, a maximum score of 10 was assigned when the DASH recommendations were achieved [21], and intakes in between were scored proportionally by a clinical research dietician (KMWM). Subjects with at least one dietary intake group received an assessment score <6 were categorised as having unhealthy diet regime. The behaviour of irregular daily meals was efined as regularly skipping meals or eating meals on an arbitrary schedule. Each interview-administered questionnaire lasted for around 20 minutes, and all questions were answered by all participants. Data entry was conducted independently by two trained university students (from The Chinese University of Hong Kong, Hong Kong), and double entry verification was performed using EpiData software version 3.1 (Denmark) [22].

### Statistical analysis

Descriptive statistics with standard deviation (SD) were used, and independent *t*-test and chi-square test were used to compare the differences between continuous variables and categorical variables, when appropriate, among four categories of hypertension (normotensive, pre-hypertensive, grade 1 hypertensive, and grade 2/3 hypertensive). Regression analysis was performed to explore factors associated with the presence of grade 1 hypertension among those having SBP ≤159 mmHg and/or DBP ≤99 mmHg. In the first step, univariate logistic regression analysis was performed and expressed as crude odds ratio (OR) with 95% confidence interval (CI). On the second step, the potential confounding effects of individual independent variables were taken into account in the multiple logistic regression model using backward stepwise algorithm, where significant variables from univariate analysis were included to evaluate independent association as adjusted odds ratio (aOR) with 95% CI. The association between the predicted probability (within the range from 0 to 1) of grade 1 hypertension (Y-axis) and the number of combined risk factors (X-axis) were plotted by gender. The absence of multicollinearity and plausible interactions among variables were tested in all regression models. Differences were regarded as statistically significant when *p* values are less than 0.05. All statistical analyses were performed by IBM SPSS Statistics 20.0 (Chicago, Illinois, US).

### Ethics statement

The study was approved by the Joint Chinese University of Hong Kong-New Territories East Cluster Clinical Research Ethics Committee, Hong Kong.

## Results

Of the total of 2,673 randomly selected subjects aged 40–70 years who received invitation phone calls, 679 subjects agreed to participate in the screening (participation rate of 25.4%, [679/2,673]), and 320 subjects were identified as having grade 1 hypertension (Figure 1). No significant differences were found between invitation respondents and non-respondents in terms of age groups (*p* = 0.414) and gender distribution (*p* = 0.389). The proportion of grade 1 hypertension and grade 2/3 hypertension were 47.1% [320/679] and 7.1% [48/679], respectively, and 32.0% [217/679] of subjects had pre-hypertension. Although there was no significant difference in average age years among subjects from four categories of hypertension (*p* = 0.332), the proportion of males increased steadily when the BP category was higher (from 19.1% [18/94] among normotensive subjects, to 66.7% [32/48] among grade 2/3 hypertensive subjects, *p* < 0.001) (Table 1).

**Table 1:** **Scio-demographic characteristics of study participants by blood pressure categories**

**Variables**	**Hypertension categories**				***p*** **value**
	**Normotensive**	**Pre-HT**	**Grade 1 HT**	**Grade 2/3 HT**	
	**(N = 94)**	**(N = 217)**	**(N = 320)**	**(N = 48)**	
**Age (years, SD)**	53.41 (2.49)	54.02 (3.45)	54.15 (4.15)	54.42 (4.02)	0.332
**Gender, %**					
Male	18 (19.1%)	83 (38.2%)	166 (51.9%)	32 (66.7%)	<0.001
Female	76 (80.9%)	134 (61.8%)	154 (48.1%)	16 (33.3%)	
**Living status, %**					
Living alone	6 (6.4%)	14 (6.5%)	6 (1.9%)	4 (8.3%)	0.022
Living with others	88 (93.6%)	202 (93.5%)	314 (98.1%)	44 (91.7%)	
**Marital status, %**					
Single	7 (7.4%)	19 (8.8%)	6 (1.9%)	3 (6.3%)	<0.001
Married	77 (81.9%)	183 (84.7%)	309 (96.6%)	42 (87.5%)	
Divorced/separated	8 (8.5%)	11 (5.1%)	4 (1.3%)	2 (4.2%)	
Widowed	2 (2.1%)	3 (1.4%)	1 (0.3%)	1 (2.1%)	
**Education level, %**					
Illiteracy	2 (2.2%)	1 (0.5%)	1 (0.3%)	1 (2.2%)	0.340
Primary school	9 (9.8%)	25 (11.8%)	41 (13.2%)	2 (4.3%)	
Middle school	14 (15.2%)	43 (20.4%)	64 (20.6%)	12 (26.1%)	
High school	47 (51.1%)	98 (46.4%)	150 (48.2%)	16 (34.8%)	
Undergraduate or above	20 (21.7%)	44 (20.9%)	55 (17.7%)	15 (32.6%)	
**Employment, %**					
Unemployed	14 (14.9%)	12 (5.6%)	61 (19.1%)	9 (18.8%)	<0.001
Employed	63 (67.0%)	166 (76.9%)	226 (70.6%)	32 (66.7%)	
Retired	17 (18.1%)	38 (17.6%)	33 (10.3%)	7 (14.6%)	
**Monthly household income, %**					
$0-$9,999	32 (41.6%)	54 (29.0%)	25 (7.9%)	7 (17.9%)	<0.001
$10,000-$19,999	17 (22.1%)	56 (30.1%)	77 (24.4%)	13 (33.3%)	
$20,000-$29,999	10 (13.0%)	33 (17.7%)	62 (19.7%)	7 (17.9%)	
$30,000-$39,999	12 (15.6%)	22 (11.8%)	86 (27.3%)	9 (23.1%)	
$40,000 and above	6 (7.8%)	21 (11.3%)	65 (20.6%)	3 (7.7%)	

HT, hypertension; SD, Standard Deviation.

In the univariate logistic regression analysis, each independent variable was evaluated for the association with grade 1 hypertension (Table 2). In the multiple logistic regression analysis, the independent associations of unhealthy diet regime (aOR = 2.19, 95% CI 1.04-4.62), irregular daily meals (aOR = 1.47, 95% CI 1.11-1.95), BMI >27.5 kg/m^2^ (aOR = 1.87, 95% CI 1.53-2.27), duration of cigarette smoking (aOR = 1.83 per year), increased daily cigarette consumption (aOR = 1.59 per pack [20 cigarettes per pack]), duration of alcohol drinking (aOR = 1.65 per year), higher frequency of weekly binge drinking (aOR = 1.87 per occasion), and the presence of parental history of hypertension (aOR = 1.08, 95% CI 1.02-1.15) with grade 1 hypertension remained statistically significant after adjusting for the potential effects of other independent factors (Table 3).

**Table 2:** **Univariate logistic regression analysis of variables associated with the presence of grade 1 hypertension**

**Independent variables**	**Presence of grade 1 hypertension**				
	**B**	**SE**	***p*** **value**	**OR**	**95% CI**
Age, 60–70 years versus 40–50 years	0.02	0.02	0.283	1.02	0.98-1.07
Gender, male	0.84	0.17	**<0.001**	2.31	1.67-3.20
Living status, alone	0.63	0.16	**<0.001**	1.88	1.38-2.55
Marital status, single	0.13	0.23	0.562	1.14	0.73-1.79
Education level, below secondary	0.08	0.07	0.207	1.09	0.96-1.23
Monthly household income, ≤$19,999	0.36	0.18	**0.045**	1.44	1.01-2.05
Unhealthy diet regime (assessment score <6)	0.25	0.12	**0.035**	1.28	1.02-1.61
Body mass index, BMI (>27.5 kg/m^2^)	0.55	0.12	**<0.001**	1.73	1.38-2.18
Hours of active physical exercises per week	0.03	0.02	0.113	1.03	0.99-1.08
Irregular daily meals on an arbitrary schedule	0.03	0.01	**0.001**	1.03	1.01-1.05
Duration of cigarette smoking, per year	0.65	0.09	**<0.001**	1.92	1.60-2.31
Daily cigarette consumption, per pack	0.05	0.02	**0.017**	1.06	1.01-1.10
Duration of alcohol drinking, per year	0.48	0.06	**<0.001**	1.61	1.42-1.82
Weekly binge drinking, per occasion	0.38	0.17	**0.025**	1.46	1.05-2.05
Presence of parental history of hypertension	1.16	0.52	**0.026**	3.20	1.15-8.91

B: Beta coefficient, SE: Standard Error, OR: Odds Ratio, CI: Confidence Interval.

Note: Cigarette consumption was measured as the average amount of cigarette consumption per day, according to the number of packs smoked per day (20 cigarettes per pack). Binge drinking of alcohol was defined as having five servings of alcohol (for males) or four servings (for females) on one occasion. Unhealthy diet regime was defined as having at least one dietary intake group with a dietary assessment score <6. The presence of irregular daily meals was defined as regularly skipping meals or eating meals on an arbitrary schedule.

**Table 3:** **Multiple logistic regression analysis of predictors of grade 1 hypertension**

**Independent variables**	**Presence of grade 1 hypertension**				
	**B**	**SE**	***p*** **value**	**Adjusted OR**	**95% CI**
Unhealthy diet regime (assessment score <6)	0.79	0.38	**0.039**	2.19	1.04-4.62
Body mass index, BMI (>27.5 kg/m^2^)	0.62	0.10	**<0.001**	1.87	1.53-2.27
Irregular daily meals on an arbitrary schedule	0.39	0.14	**0.008**	1.47	1.11-1.95
Duration of cigarette smoking, per year	0.60	0.26	**0.018**	1.83	1.11-3.02
Daily cigarette consumption, per pack	0.46	0.12	**<0.001**	1.59	1.24-2.02
Duration of alcohol drinking, per year	0.50	0.17	**0.004**	1.65	1.18-2.33
Weekly binge drinking, per occasion	0.62	0.27	**0.023**	1.87	1.09-3.20
Presence of parental history of hypertension	0.08	0.03	**0.015**	1.08	1.02-1.15

B: Beta coefficient, SE: Standard Error, OR: Odds Ratio, CI: Confidence Interval.

Note: Logistic regression model includes terms of unhealthy diet regime (dietary assessment score <6), BMI (>27.5 kg/m^2^, high risk versus 18.5-22.9 kg/m^2^, increasing but acceptable risk), presence of irregular meals (regularly skipping meals or eating meals on an arbitrary schedule), duration of cigarette smoking and daily consumption (per pack [20 cigarettes per pack]), duration of alcohol consumption and frequency of weekly binge drinking (per occasion [five servings of alcohol for males or four servings for females on one occasion]), and parental history of hypertension (presence versus absence), based on backward stepwise algorithm selection from variables that were significant in the univariate analysis.

**Figure 2:**
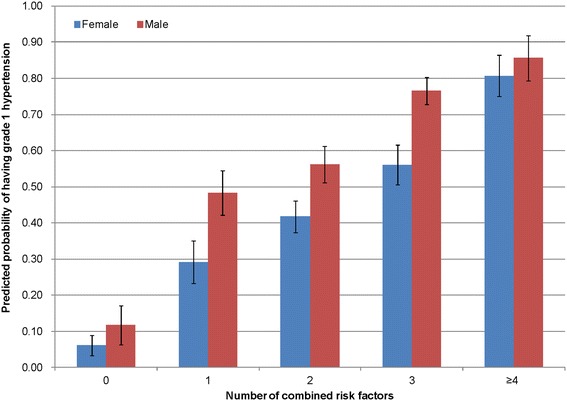
**Number of risk factors and predicted probability of grade 1 hypertension by gender.**

The logistic regression analysis also showed that the increased number of combined risk factors was significantly associated with a higher predicted probability of having grade 1 hypertension in both males and females, albeit the extent of such increases observed seemed to be more striking in the female group. Among male subjects, those with four or more combined risk factors had seven times the predicted probability of having grade 1 hypertension when compared to subjects with no risk factors (0.86, 95% CI 0.78-0.93 versus 0.12, 95% CI 0.05-0.18). Whereas among female subjects, individuals with four or more combined risk factors had approximately thirteen times the predicted probability of grade 1 hypertension when the number of combined risk factors increases (0.81, 95%CI 0.74-0.87 versus 0.06, 95% CI 0.03-0.09) (Figure 2).

## Discussion

### Statement of principal findings

From a community-based population sample aged 40 to 70 years enrolled in a primary care programme, almost half of the subjects were grade 1 hypertensive. The study found that unhealthy diet regime (having dietary assessment score <6 with reference to the DASH recommendation), high risk BMI (>27.5 kg/m^2^), irregular daily meals on an arbitrary schedule, cigarette smoking, alcohol drinks, and the presence of parental history of hypertension were independently and significantly associated with grade 1 hypertension. The increase in the number of combined risk factors also significantly correlated with higher predicted probability of grade 1 hypertension. Subjects with four or more combined risk factors had seven times (for males) and thirteen times (for females) the predicted probability of having grade 1 hypertension when compared to subjects with no risk factors.

### Relationship with other studies

The burden of hypertension-related chronic conditions in Hong Kong has been increasing in recent years [23], while complicated hypertension might lead to increased risk of multimorbidity [24-28]. However, few studies focused on the prevalence and determinants of grade 1 hypertension (an early phase of disease progression to complicated hypertension) in primary care settings. Literature has reported the prevalence or diagnosed rate of grade 1 hypertension in the population worldwide. In a national representative sample of non-institutional population in England, 21.5% of subjects aged 40–79 years were on-site diagnosed with grade 1 hypertension using automatic oscillo-metric devices [29]. In Korea, a population-based cross-sectional survey with BP measurement in the field among 4,226 adults aged 19–92 years revealed that the overall prevalence of hypertension was 33.7%, among which, 64.9% had grade 1 hypertension [30]. Another integrated community-based screening programme in Taiwan reported a prevalence of 34.4% individuals having grade 1 hypertension among 39,542 subjects aged 40-70 years [31]. In our study in Hong Kong, nearly half of participants had grade 1 hypertension, which was slightly higher than previous studies. It is plausible that the study participants were more health conscious subjects who tended to consider themselves as having a higher risk of high BP prior to attending the onsite screening, and thus reflected a higher proportion observed. Despite the varying degrees of disease prevalence, grade 1 hypertension in general should receive more attention in primary care particularly among those untreated patients, as most ischemic strokes could occur at this stage [32].

This study did not directly assess the awareness of grade 1 hypertension among study participants; however, a considerably low participation rate was observed in the screening. This could partially reflect the current situation that people might have less awareness of the importance of elevated BP and its potential consequences. Studies have suggested that raised BP often showed no clear symptom which could be easily missed out by patients, and this often led to inadequate awareness of hypertension [33,34]. The Reference Framework advocated active BP screening among the general population [4], yet the inferior participation rate in the BP screening observed in this study may imply a need for improving health system-based strategies to encourage screening participation [35]. As primary care plays a pivotal role in the healthcare system [4,36-41], suggested local solutions might include the provision of regular screening programme by primary care providers particularly in private healthcare sectors where it accounted for approximately 70% of clinic consultations in total [42]. The establishment of long-term community-based multi-sectoral education campaign for participation motivation particularly among those who are at higher risk of grade 1 hypertension could also be of importance. It has been suggested that community-based preventive measures at population level could largely decrease the morbidity and mortality of cardiovascular and cerebrovascular diseases [43] which are major adverse outcomes of hypertension at a later stage. In other developing regions worldwide, the implementation of programmes using top-down and bottom-up approaches to identify hypertensive patients and raise their awareness on the diseases in an early stage also demonstrated the effectiveness in hypertension control [44,45].

An earlier systematic review on the crude prevalence of hypertension among adults worldwide showed non-significant gender differences [46], and our study also showed that the increase in the number of combined risk factors significantly correlated with higher predicted probability of grade 1 hypertension in both males and females. The extent of such increases observed seemed to be more striking in the female group, after controlling for other socio-demographics and lifestyle factors, and reasons for this phenomenon are yet to be explored. Some early evidence on cardiovascular risks demonstrated that the lipoprotein(a) may seem to play a role in the different gender effect [47,48]; however, the level of lipoprotein was not assessed in our study. Further exploratory studies might be needed to assess the different responses to risk factors for grade 1 hypertension in men and women respectively, and this should be acknowledged in the management and interventional strategies of grade 1 hypertension.

It has been shown that for uncomplicated grade 1 hypertension, efficient lifestyle modifications and initiatives could play a role [49-51]. The Reference Framework [10] recommended lifestyle approaches rather than pharmacological interventions for the management of grade 1 hypertension, including advices on increasing the consumption of fruits and vegetables while reducing total and saturated fat consumption. The study findings that lifestyle factors such as unhealthy diet regime and irregular daily meals contributed to the presence of grade 1 hypertension could support the integration of the DASH approaches in the Reference Framework, which was developed to promote a diet regime emphasised fruits, vegetables, and low-fat dairy products [11]. Previous literature documented a high and increasing prevalence of hypertension in patients with suboptimal body weight control [52], and in our study it was observed that subjects of high-risk BMI group had the highest adjusted odds ratio of developing grade 1 hypertension. This further suggested the importance of DASH regime, which was shown to significantly reduce weight in western population [11,12,53,54]. It may also imply the need for developing specific tailor-made programmes on weight reduction in parallel with the adoption of DASH approaches to benefit those who are at higher risk of suboptimal level of healthy diet intake and increasing body weight.

### Strengths and weaknesses of the study

From a public health perspective, this study illustrated the initiative to implement the Reference Framework for Hypertension Care into action to screen grade 1 hypertensive subjects in primary care settings in Hong Kong where the prevalence of hypertension is increasing. The risk factors explored in the study could inform policy strategies on contextualising health policies and developing primary care-based grade 1 hypertension intervention modules in addition to the DASH approach. From a family practice perspective, the study provided information for frontline primary care practitioners to design tailor-made care strategies for those who are at higher risk of grade 1 hypertension in routine clinic.

There were several limitations of the study. Firstly, the BP measurement was performed during one diurnal visit in the clinic onsite which might incur white-coat effect, and the absolute cardiovascular risk was not calculated as blood glucose and lipids parameters [55] were not measured. Secondly, it is possible that those who agreed to participate in the screening were more health conscious or had higher rates of certain risk factors as identified in the study, compared to non-respondents whose information on these risk factors were unknown due to their non-participation in the interviewer-administered survey. This may affect the disease prevalence and skew the association between risk factors and grade 1 hypertension observed in this study, and thus the results should be interpreted with caution. Thirdly, the study only included subjects whose ages were within the range of 40–70 years, which might limit the external applicability of the study results. However, subjects within this age group were considered having doubled risk of cardiovascular diseases for each increment of 20 mmHg in SBP or 10 mmHg in DBP [6] respectively, and thus they should be given considerable attention in primary care. Fourthly, recall bias might exist in the information on lifestyle characteristics and parental medical history which were collected from patient’s self report. Last but not least, the cause-and-effect relationship was unable to examine due to the cross-sectional nature. Further follow-up DASH dietary intervention incorporated with risk factors identified in this study may provide more evidence to inform primary care strategies for grade 1 hypertension in the community.

## Conclusions

This cross-sectional study explored the association between grade 1 hypertension and quantified lifestyle risk factors. Unhealthy diet regime and irregular daily meals were associated with grade 1 hypertension, echoing the recommendation in the Reference Framework on incorporating dietary intervention based on the DASH approach for hypertension care in primary care settings. The higher predicted probability of grade 1 hypertension due to greater numbers of combined risk factors should be taken into consideration in future long-term preventive approaches. In addition, the provision of regular screening programme by primary care providers might be needed in parallel with the implementation of DASH intervention on grade 1 hypertensive subjects in the community.

### Availability of supporting data

The authors confirm that all data underlying the findings described in this manuscript is fully available to all interested researchers upon request. The study involved human participants, and requests should be submitted to the Joint Chinese University of Hong Kong-New Territories East Cluster Clinical Research Ethics Committee, Hong Kong [Study DOI: CRE-2010.372].

## Abbreviations

DASH: Dietary approaches to stop hypertension
BP: Blood pressure
SBP: Systolic blood pressure
DBP: Diastolic blood pressure
BMI: Body mass index
